# Optimization of airport check-in service quality focused on operational costs and passengers’ satisfaction

**DOI:** 10.1371/journal.pone.0253586

**Published:** 2021-08-05

**Authors:** Ludovica Adacher, Marta Flamini

**Affiliations:** 1 Department of Engineering, Roma Tre University, Rome, Italy; 2 Faculty of Engineering, International Telematic University UNINETTUNO, Rome, Italy; Sichuan University, CHINA

## Abstract

Passengers’ requirements in relation to the Airport Service Quality is rapidly increasing and forcing companies and airport management to improve the services performances. It is clear that this enhancement can not overlook the implication of competitive issues and economic concerns. In this paper the authors deal with the optimization of the check-in area management in the international airport of Lisbon. The proposed bi-criteria objective function minimizes the operational costs plus the costs measuring the passengers’ discomfort in terms of waiting time in line. The quality of the supplied check-in service is measured and mapped into the Levels of Service system standardized by the International Air Transport Association. The type of passengers and their stochastic behavior and preferences are simulated by a discrete event model. The operational costs and the passengers’ satisfaction are optimized by an algorithm based on the Surrogate Method, the performance of which are compared to those of a greedy heuristic and of a genetic algorithm.

## 1 Introduction

Airport land side processes involve several types of services and resources, the performance of which affects both the costs incurred by the stakeholders and the passengers’ satisfaction [1]. For this reason, Airport Service Quality (ASQ) has become a crucial issue for all the agents involved in airport operations [2]. It regards all the services supplied in the airport and affects many performance indices which imply economic issues and competitiveness concerns. The ASQ depends on service delivery (frequently performed by human operators) and on passengers’ perception [3–5]; hence models representing, and/or predicting the stochastic behavior of the system agents, become important to address the problems concerning the resources optimization in a realistic way [6]. In addition, the ASQ calls for models and algorithms able to represent, measure and optimize the performed and perceived quality of service. In fact, one of the main research challenges consists of studying and modelling the complex connection among the service performance, the passengers perception and the ASQ levels [7–11]. These representations should be integrated in the models that describe the complex system of the airport, where several resources, services and passengers types interact and where the optimization of many key performance indices is required. One of the most critical airport land side operation is represented by the check-in service. Recently smart airports have implemented fast self service solutions such as web service and kiosks. Nevertheless the traditional check-in procedures are still preferred when dropping off the baggage, in case of unexpected events, or by middle-aged people. The optimization of the check-in operations has been addressed by many authors. The first papers on the check-in problem are based on simulation approaches. Only in the last decades optimization or hybrid approaches have been introduced. However, many papers highlight that an efficient use of check-in counter operations can be achieved understanding passengers’ behavior and preferences [12–14]. The necessity of modelling passengers behavior and optimizing check-in operational costs calls for the integration of simulation models and optimization techniques to address such problems. Some authors focus on minimizing the number of counters required for daily operations others focus on minimizing the waiting times of passengers [15–19]. In [20], the authors address the problem of optimizing the number of check-in desks to be activated and the waiting time of the passengers. In [21] the authors improve the previous model by introducing the shifts/breaks schedules for the employers and minimize the personnel costs at the check-in desks. They also bound the maximum waiting time of passengers by parametric constraints that limit the length of the lines in a time interval to a given percentage of the arrival flow in the same time interval. In [16] the authors optimize the opening of different types of check-in by considering the passengers flow as demand for a certain type of service. In [22] the authors introduce service level constraints in the problem of optimizing the check-in allocation and validate the model providing results by simulation. Hence the procedure also takes into account the service quality perceived by the passengers [23]. Several authors focus on the minimization of the waiting time of passengers requesting the check-in services by implementing bi-criteria approaches that take into account the maximum satisfaction and the cost minimization. They provide the optimization of a bi-criteria cost function that involves the costs of opening check-in counters and the costs modelling passengers’ waiting time. The evolutionary algorithms are the most used technique to solve this multi-criteria problem, due to its complexity. Many papers [24, 25] solve similar problems by using commercial simulators [26–28] provided with embedded optimization tools that are mainly based on genetic heuristics.

In this paper the authors optimize the activation of the check-in counters focusing on operational costs and passengers’ satisfaction. With this aim, the objective considers both the minimization of the operational costs and the maximization of the passengers’ satisfaction. The costs refer to the personnel cost at the check-in desks, while the passenger satisfaction is related to the waiting time in line. In particular, the combination of simulation and optimization techniques is proposed to solve the problem of assigning check-in counters to the flights in a given time interval. The interaction between simulation and optimization phases provides the objective function value and its minimization. The simulation module is implemented and the optimization of the check-in service quality is performed by the Surrogate Method Heuristic (SM) [29]. It is a complex procedure that easily avoids local minima of the objective function. To validate the effectiveness of the solutions produced by the *SM*, two heuristic procedures have been implemented, namely a greedy heuristic improved by a local search and a genetic algorithm. The greedy procedure easily suites the quality aspect of the problem while the genetic algorithm performance constitutes a benchmark since it is embedded in several simulation tools. The aim of the paper consists in analysing and comparing the *SM* results to the other two procedures. As for the service quality several authors derive the passengers’ satisfaction by analysing historical data related to passengers’ behavior. They define the service quality function by using statistical data collected by the airline companies or by the airport management through questionnaires [2, 3, 6, 7]. In this paper the service quality has been modeled by a discomfort cost function directly derived by the International Air Transport Association (IATA) standards [32]. Hence the effectiveness of the computed solutions can be easily mapped into the IATA service quality levels.

Authors refer to the case study of Lisbon airport where realistic passenger flow and real air traffic schedule have been simulated. This case study has been considered to test the effectiveness of *SM*, without loss of generality since it can be adapted and applied to any terminal area.

The main contributions of this study are the following:

- a bi-criteria cost function is considered, balancing the check-in counters operational costs and the passengers discomfort based on the IATA standards;
- an optimization/simulation decision support system has been implemented;
- the Surrogate Method Heuristic has been properly adapted to the addressed problem and its performances have been compared to greedy and genetic algorithms;

The paper is organized as follows: Section 2 presents the problem description. In section 3 the methodology is reported, where an optimization module, described in 3.1 cyclically interacts with a simulation module, described in 3.2. The optimization module embeds one of the three heuristic procedures presented in the paper. The simulation module models the different types of passengers and their stochastic behavior when lining up. In Section 4 tests are described and results are reported and analyzed. Section 5 is dedicated to the conclusion and to the description of possible future developments of the study.

## 2 Problem description

In this section the problem formulation is provided and the cost function is presented. A set of flights *F*, a set of check-in counters *C* located in a terminal, and a time interval of *T* consecutive time slots are the input of the problem. Each check-in counter is associated to a single company; hence it is assumed that the Check-in assignment problem has been already solved [30, 31]. usually a check-in desk can be *common* or *dedicated*. A common check-in counter can serve passengers of different flights of the same airline company, while a dedicated check-in counter processes only the passengers of a specific flight. Each flight *f* ∈ *F* has associated a take off time *t*_*o*_ *ff*^*f*^ and a departure time slot $t_{d}^{f}$, with *t*_*o*_ *ff*^*f*^ ∈ *T* and *t*_*o*_ *ff*^*f*^ ∈ *T*. The maximum number of check-in counters that can be open in a time slot is *C*_*max*_, that corresponds to the total number of check-in desks that are located in the check-in area of the terminal. *Q*_*max*_ is the maximum number of passengers that can wait in line due to the spatial constraints. This paper focuses on the problem of assigning the check-in counters to the flights with the objective of minimizing a cost function which takes into account the personnel costs, associated to the opening of the desks, and the passengers dissatisfaction, depending on the length of lines. Hence, the passengers satisfaction is maximized by minimizing the waiting time in line.

The variables of the problem can be divided into optimization variables and simulation variables:

The optimization variables are decision variables and can be expressed as:

$$y_{ct}=\{\begin{array}{ll} 1 & \text{if}\;\text{the}\;\text{check-in}\;\text{counter}\;c\;\text{is}\;\text{active}\;\text{in}\;\text{the}\;\text{time}\;\text{slot}\;t\\ & \text{for}\;\text{c}=1\ldots |C|,\;\text{t}=1\ldots |T|\\ 0 & \text{otherwise} \end{array}$$ (1)

$$x_{fct}=\{\begin{array}{ll} 1 & \text{if}\;\text{flight}\;f\;\text{is}\;\text{associated}\;\text{to}\;\text{the}\;\text{check-in}\\ & \text{counter}\;c\;\text{in}\;\text{the}\;\text{time}\;\text{slot}\;t\\ & \text{for}\;\text{f}=1\ldots |F|,\;\text{c}=1\ldots |C|,\;\text{t}=1\ldots |T|\\ 0 & \text{otherwise} \end{array}$$ (2)

The simulation variables values depend on the decision variables and are evaluated by the simulation module. The simulation variables are:

- *q*_*ct*_ which indicates the number of passengers in line at the check-in desk *c* during the time slot *t*;
- *wt*^*p*^ which indicates the waiting time of passenger *p* in line (in minutes).

In fact, because of the stochastic behavior of the passengers moving in the check-in area, the construction of a feasible solution must be completed by a simulation module that models the passenger arrangement in lines. It provides information about the waiting time of each passenger in line and the length of lines at each check-in counter. This link is detailed in section 3 where the methodology is described as the interaction between the optimization and simulation modules.

The objective function expression is reported in Eq 3 and combines the operational costs and the passengers’ waiting time cost. More specifically, *C*_*o*_ represents the unit cost of opening a check-in desk for a time slot and *C*_*p*_(*wt*^*p*^) represents the cost of the passenger *p* waiting in line *wt*^*p*^ minutes.

$$\begin{array}{c} OF=\text{min}\sum_{c=1}^{C}\sum_{t=1}^{T}C_{o}y_{ct}+\sum_{p=1}^{P}C_{p}\left(wt^{p}\right) \end{array}$$ (3)

**Fig 1:**
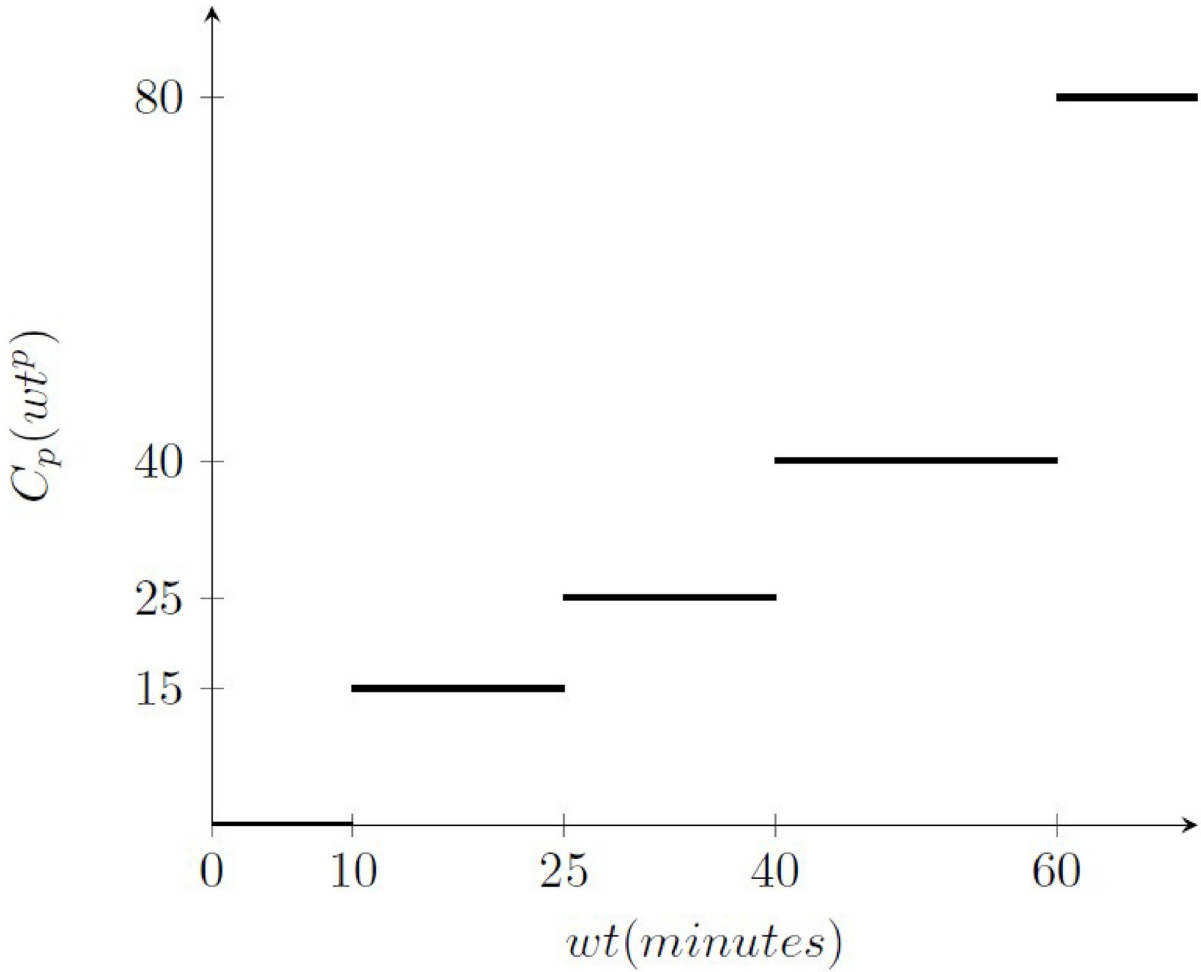
Cost function depending on the waiting time.

The function *C*_*p*_(*wt*^*p*^) is a step function that depends on the waiting time in line. Its trend is depicted in Fig 1. From now on the notation *C*_*o*_ is used for operational cost and *C*_*p*_(*wt*^*p*^) is used for passengers discomfort cost. The total cost function is given by the sum of the two previous described cost functions. It guarantees a good trade-off between the two terms of the objective function.

The constraints of the problem are the following:

1. ∑_*c*∈*C*_ *y*_*ct*_ ≤ *C*_*max*_ for each *t* ∈ *T*
2. *q*_*ct*_ ≤ *Q*_*max*_ for each *c* ∈ *C*, for each *t* ∈ *T*
3. ∑_*c*∈*C*_ ∑_*t*∈*T*_ *x*_*fct*_ ≥ 1 for each *f* ∈ *F*
4. ∑_*f*∈*F*_ *x*_*fct*_ ≥ *y*_*ct*_ for each *c* ∈ *C*, for each *t* ∈ *T*
5.
$\sum_{c\in C}\sum_{t=t_{d}^{f}-s-1}^{t_{d}^{f}-1}y_{ct}\geq s$ for each *f* ∈ *F*
6.
$\sum_{c\in C}\sum_{t=t_{d}^{f}-s-1}^{t_{d}^{f}-1}x_{fct}\geq \sum_{c\in C}\sum_{t=t_{d}^{f}-s-1}^{t_{d}^{f}-1}y_{ct}$ for each *f* ∈ *F*
7.
$\sum_{c\in C}\sum_{t=1}^{t_{d}^{f}-s-2}x_{fct}=0$ and

$\sum_{c\in C}\sum_{t=t_{d}^{f}}^{T}x_{fct}=0$ for each *f* ∈ *F*

The meaning of each constraint is explained in the following, where the reference numbers relate to the expressions reported above:

1. the maximum number of open check-in counters in each time slot is limited to *C*_*max*_;
2. the length of each line is limited to *Q*_*max*_;
3. each flight must be assigned to at least one check-in desk;
4. a check-in counter is activated only if at least one flight is associated to it;
5. the check-in operations must last *s* consecutive time slots for each flight *f* and must end one slot before $t_{d}^{f}$;
6. each flight has to be assigned to at least one open check-in desk;
7. flight *f* has associated a check-in desk only in the interval $\left[t_{d}^{f}-1-s,t_{d}^{f}-1\right]$.

Constraints 1 and 2 refer to capacity constraints of the check-in area, while constraints 3–7 can be addressed as service constraints.

In this paper, the service quality of a solution is evaluated in terms of Level of Service (LOS) perceived by the passenger. The evaluation of the perceived level of service has been standardized by the IATA [32]. The levels correspond to Excellent (A), High (B), Good (C), Adequate (D) and Inadequate (E). In Table 1 the relation between the Level of Service and the maximum tolerable waiting time in line (in minutes) are reported.

**Table 1:** Levels of service and corresponding waiting times (min) [32].

Level of Service	A	B	C	D	E
Max waiting time	10	25	40	60	≥ 61

Thanks to this correspondence, each computed solution can be evaluated also in terms of standard LOS.

## 3 Methodology

**Fig 2:**
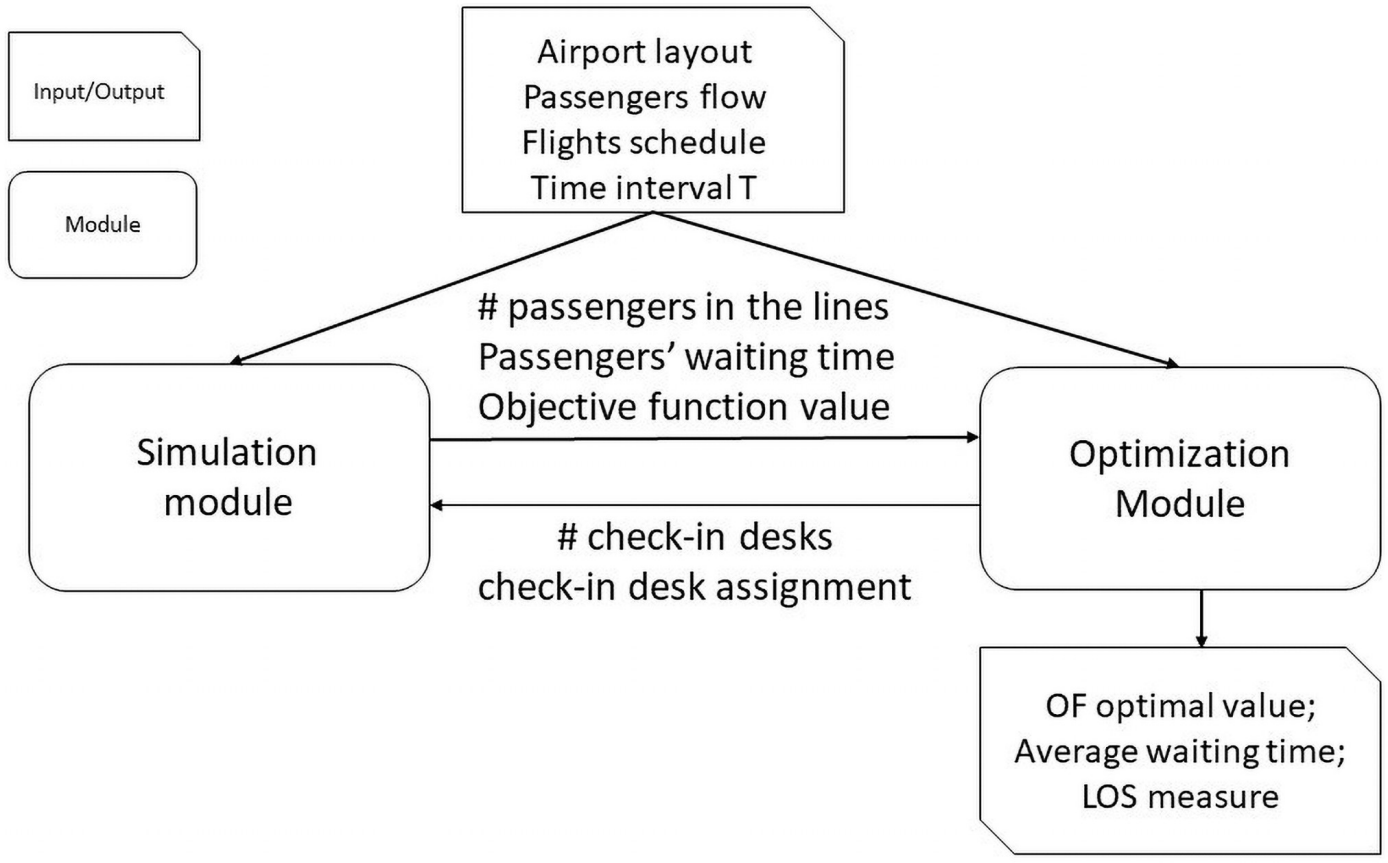
Integration of the simulation module with the optimization module.

In this section the optimization and simulation modules are described. In Fig 2 the interaction between the two modules is depicted. The input to the system is the airport layout (area capacity constraints), the passengers flow and the flights schedule in a given time interval. The simulation module arranges the passengers in lines in a rationale way: it models and considers their needs, types, preferences and behavior. It transfers the information about the objective function value and the passengers’ waiting time to the optimization module. The optimization module optimizes the number of the check-in counters and their assignment to the flights. The two modules cyclically interact until a stop criterion is verified. The output of the system consists of the best value of the objective function, the associated average passengers’ waiting time and the measure of the Level of Service.

### 3.1 Optimization module

The optimization module embeds an optimization algorithm. Three different algorithms have been tested considering the same input data sets. As already mentioned, the three procedures are: a Surrogate Method Heuristic, a greedy algorithm improved by a local search procedure and a standard genetic algorithm. This paper focuses on the effectiveness of the Surrogate Methdod Heuristic that can be validated by comparing its performance to those of the other two procedures.

In the following the three procedures are described and the related pseudocode is reported.

#### 3.1.1 The Surrogate Heuristic

The optimization procedure based on the Surrogate Method Heuristic (*SM*) is reported in Algorithm 1. The *SM* was firstly studied by Gokbayrak and Cassandras [29], to solve stochastic discrete optimization problems with no negative integer decision variables. The *SM* proves to be effective in various application areas returning good or sub-optimal solutions to the original discrete problem and assuring very fast convergence [33, 34]. In two previous works [15, 35] the authors applied the *SM* to a similar problem with a single airline company and some restrictions on passenger categories.

The *SM* solves a discrete problem by (i) relaxing the integer constraints, (ii) solving the continuous problem using the gradient estimation in the discrete field, (iii) mapping the continuous solutions in the discrete domain, (iv) choosing the best discrete solution. The *SM* presents an iterative structure that, at each cycle, transforms the discrete problem with decision variable vector *z*, into an optimization problem with continuous decision variable vector *ρ*, by relaxing the integer constraints. The latter problem is denoted as surrogate. Let *A*_*d*_ be the sets of feasible discrete solutions for the discrete problem. Analogously, let *A*_*c*_ the set of feasible solutions for the surrogate (continuous) problem. The gradient estimate of the objective function (∇*OF*), which is used to update the solution, is computed in the discrete field. The transition from the discrete problem to the continuous problem occurs at each cycle of the algorithm and the update of the surrogate solution is obtained by considering the variation of the objective function computed in the discrete state.

In Algorithm 1 the reader can find the steps of the algorithm on the left side and the comments on the right side. The comments in bold represent the main phases of the algorithm. Parameters *H* and *K* represent the maximum number of consecutive iterations if the solution does not improve, and the maximum number of iterations of the algorithm, respectively.

Vector *z* is an integer *M*-dimensional decision vector where each component denotes the number of counters that have to be activated for each of the *M* airline companies in a specific time slot *t*, subject to the area capacity constraints. More in detail, if the first airline company has associated 10 check-in desks (from 1 to 10) and the first component of vector *z*, in a specific time slot *t*, is equal to 3 (*z*[1] = 3) it means that the number of check-in desks serving the company is equal to 3. Moreover, with regards to the formulation described in Section 2, variables *y*_*ct*_ are set to *y*_1*t*_ = *y*_2*t*_ = *y*_3*t*_ = 1, *y*_4*t*_…*y*_10*t*_ = 0. *OF*(*z*) denotes the cost when the discrete solution is *z*.

Note that the sequence {*ρ*_*k*_}, *k* = 1, 2, …, generated by an iterative scheme to solve the relaxed problem, consists of real-valued solutions which are unfeasible for the discrete problem. Thus, a key feature of the *SM* is that at each iteration *k* of the scheme, a discrete state *z*_*k*_ is updated through the mapping function *f*: *z*_*k*_ = *f*(*ρ*_*k*_). Function *f* selects the integer *z*_*k*_ that minimizes the difference |*z* − *ρ*_*k*_| as reported in step 4.

This procedure has two main advantages:

- the cost of the discrete system is adjusted at each cycle (in contrast to an adjustment that could be implemented only at the end of the surrogate optimization process);
- the scheme combines the advantages of the stochastic approximation of the algorithm with the ability to obtain sensitivity estimates in the discrete problem. In fact, in the steps 15 and 16 of Algorithm 1, the update of the continuous state is obtained by calculating the gradient of the objective function of the problem solution with an integer solution.

**Algorithm 1** The Surrogate Heuristic

1: Initialize *ρ*_0_ = *z*_0_ satisfying constraints 1.-7.  ▹ *ρ*_0_ is a continuous vector, *z*_0_ is a discrete vector,

                    ▹ both of dimension *M*

2: Initialize ρ* = *ρ*_0_, z* = *z*_0_  ▹ ρ* is the best solution of the continuous problem

3: Initialize *h* = 0, *k* = 0

4 Let $f\left(\rho_{j}\right)=z=\text{arg}\;{\text{min}}_{z\in A_{d}}\|z-\rho \|$  ▹ Transformation function *f* from the  ▹ continuous solution *ρ* to a discrete solution *z*

5: **while** ((*k* ≤ *K*) ∨ (*h* ≤ *H*))**do**

             ▹ **Form the selection set** *S*(*ρ*_*k*_) (steps 5–13):

6: Initialize I = {1, …, *M*} and *v* = *ρ*—⌊*ρ*⌋

7: **while** *I* ≠ ∅ **do**

8:  *i* = arg min_*j*∈*I*_ (*v*[*j*])  ▹ the component *v*[*i*] is the decimal part of the *ρ*[*i*] component

9:  *y*[*i*] = *v*[*i*]

10:  *W*_*i*_ = ∑_*j*∈*I*_ *e*_*j*_

11:  *v* = *v* − *y*[*i*]*W*_*i*_

12:  *I* = *I*\{*i*}

13: **end while**

14: *S*(*ρ*_*k*_) = {*W*_*i*_ − ⌊*ρ*⌋, *i* = 0, ‥, *M*} = {*s*_0_, *s*_2_, …, *s*_*M*_}

                      ▹ **Gradient estimate**

15: ∇*OF*(*ρ*_*k*_) = [∇_1_ *OF*, …., ∇_*M*_ *OF*]^*T*^        ▹ *OF* declared in (3),

                   ▹ where ∇_*j*_ *OF*(*ρ*_*k*_) = *OF*(*p*) − *OF*(*q*)

               ▹ where *k* satisfies *p* − *q* = *e*_*j*_ and *p*, *q* ∈ *S*(*ρ*_*k*_),

           ▹ being *e*_*j*_ the versor with *j*-th component equal to 1

                        ▹ **State update**

16: *ρ*_*k*+1_ = *ρ*_*k*_ − *η*_*k*_∇*OF*(*ρ*_*k*_)

17: *z*_*k*+1_ = *f*(*ρ*_*k*+1_)

18: *k* = *k* + 1

                    ▹ **Best solution update**

19: **if** *OF*(*ρ*_*k*+1_) ≤ *OF*(ρ*) **then**

20:  ρ* = *ρ*_*k*+1_

21:  *h* = 0

22: **else**

23:  *h* = *h* + 1

24: **end if**

25: **if** OF(*z*_*k*+1_) ≤ *OF*(z*) **then**

26:  z* = *z*_*k*+1_

27: **end if**

28: **end while**

                  ▹ **Return the best solution** z*

29: **Return** z*

**Fig 3:**
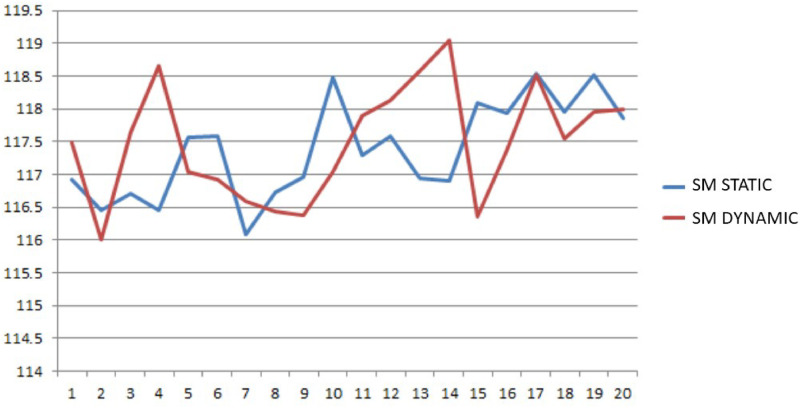
Objective function trend with static and dynamic step size.

**Fig 4:**
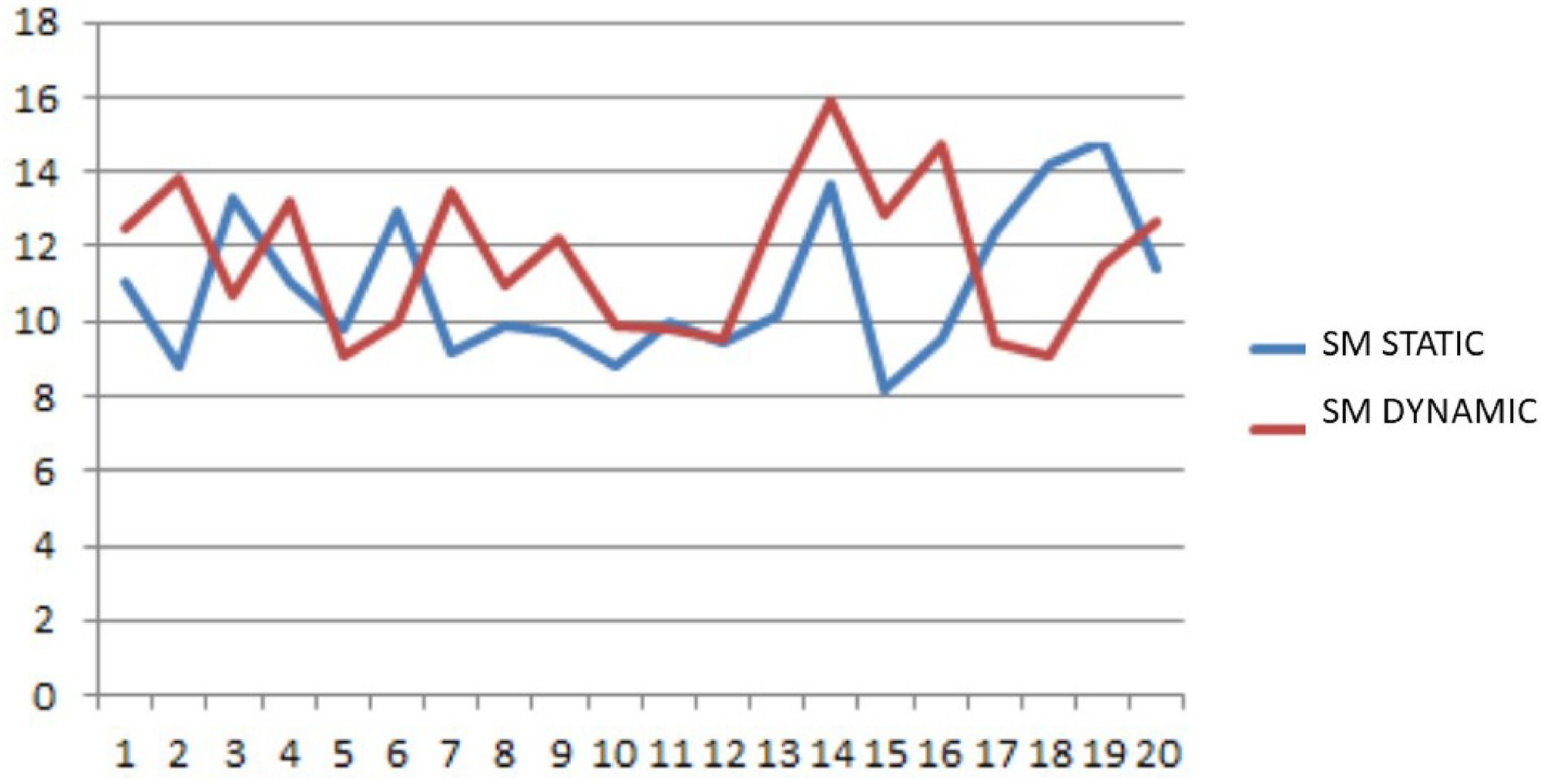
Mean waiting time trend with static and dynamic steps.

In many real problems the convergence of the *SM* depends on the step size *η* (step 16). For this reason a comparison between static and dynamic steps has been implemented. The static step means that the step is constant for each iteration, while the dynamic step changes in relation to the value assumed by the gradient in each iteration. Obviously the dynamic step needs higher computational time to be updated. In Figs 3 and 4 the trend of the objective function and of the waiting time, related to 20 problem instances, for the two different step size methods is reported. In both cases the two patterns present an oscillatory behavior and many local minimum points. Such feature makes the problem hard to be solved and calls for a specific algorithm properly designed to jump out of the local minimum points. This is a noteworthy feature of the *SM*. Nevertheless, in this case the dynamic step does not produce considerable benefits. Hence, to limit the computation time, the static step case has been implemented.

**Fig 5:**
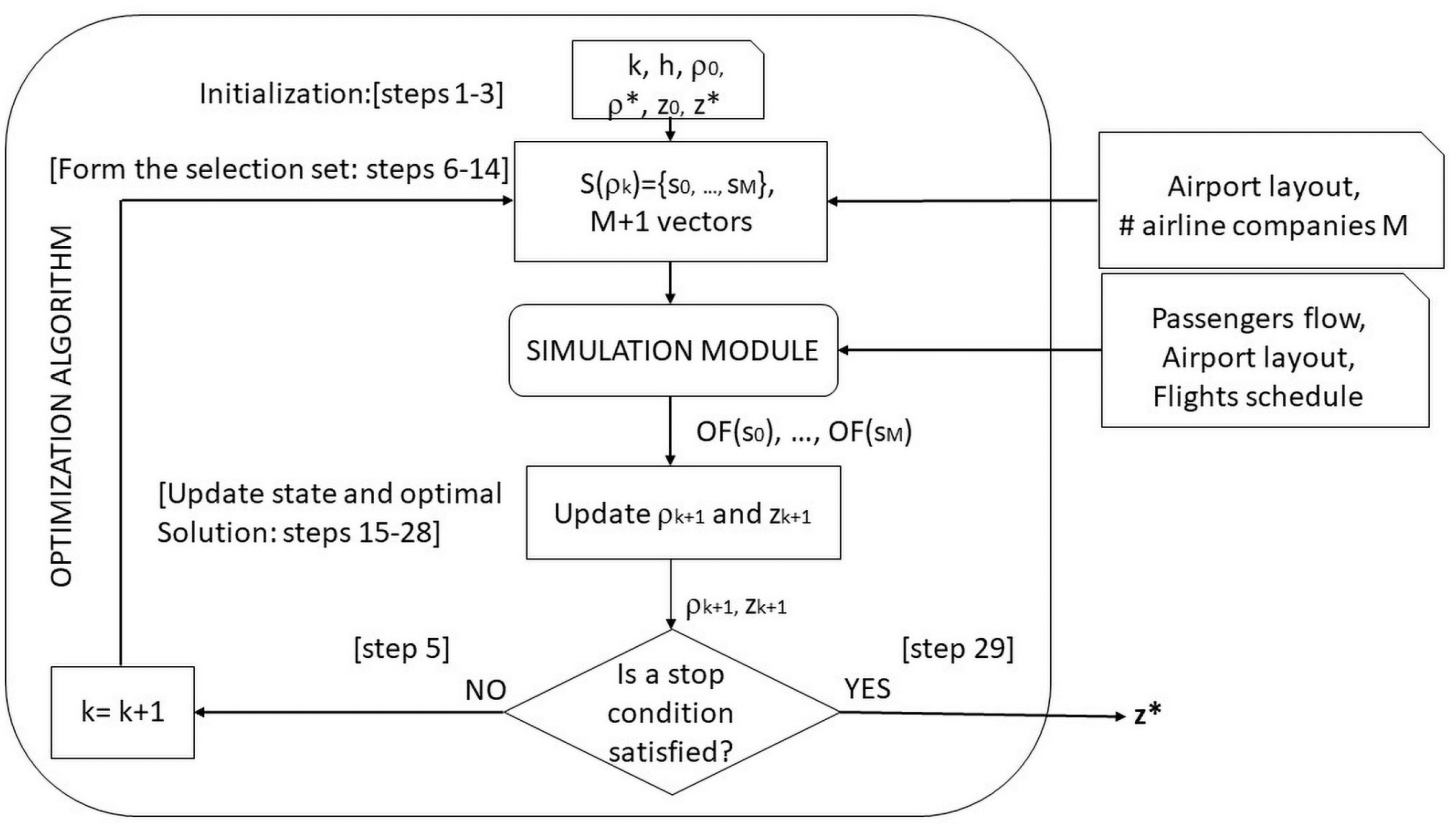
Integration of the simulation module with the Surragate Heuristic.

In Fig 5 the integration of the simulation module into the optimization module is now detailed. The Surrogate Heuristic starts from a random feasible solution and determines the selection set *S*(*ρ*_*k*_) described in Steps 5–13 of Algorithm 1.

Notice that, for each updating step of the surrogate state (step 16 of Algorithm 1) the objective function value is computed *M* + 1 times by the simulation module. Hence, it becomes crucial to develop an efficient simulation module which performs an acceptable computation time. Then the best solutions, both continuous and discrete are updated and the system cycles until a stopping criterion is not met. The output of the system is z*, that is the discrete best solution.

#### 3.1.2 Greedy heuristic + local search

The greedy procedure is very simple and fast. It constructs a solution so that the service quality of the check-in area can be maximized. Before briefly describing the main steps of the greedy procedure, the definition of the set *X*_*f*_, associated to the flight *f*, is provided. *X*_*f*_ is composed by the flights of the same airline of *f*, such that there is an overlapping of their check-in operations with those of *f* (see constraint 5 of the formulation in 2). The heuristic can be summed up in the steps reported in Algorithm 2. It is composed by two phases, the first implementing the greedy procedure and the second executing a local search procedure. The greedy phase tries to assign a set of check-in counters to each flight, so that the maximum length of the lines is under a tolerable threshold *K*. The local search phase tries to improve the solution computed in the first phase. It is trivial to infer that the solutions produced by the algorithm rarely exceed the tolerated waiting time.

**Algorithm 2** Greedy heuristic and Local search

1: Given *F*           ▹ Set of flights

2: Given *C*_*m*_ *ax*     ▹ Total number of the check-in counters

3: Given *K*        ▹ *K* is the maximum length of line

4: *C*_*ass*_ = 0    ▹ Counter: it counts the number of assigned check-in desks

5:           ▹**Greedy heuristic phase**

6: Sort *F* by increasing departure times

7: **for** (each *f* ∈ *F*) **do**

8:  Generate *X*_*f*_; ▹ *X*_*f*_ is the set of all the flights *f*′ ∈ *F* of the same airline, such that *τ*_*f*_ and *τ*_*f*′_ overlap

9: **end for**

10: **for** (each *f* ∈ *F*) **do**

11:  Assign to *f* a check-in counter list *CKC*_*f*_, one every *K* passengers;

12:  *C*_*ass*_ = *C*_*ass*_ + |*CKC*_*f*_|;   ▹ Total number of check-in counters assigned till *f*

13:  **while** (*C*_*ass*_ > *C*_*max*_) **do**

14:   Remove a check-in counter from the list

15:   of a previous flight *f*′ such that *f*′ ∈ *X*_*f*_

16:   and |*CKC*_*f*′_| ≥ 2;

17:   *C*_*ass*_ = *C*_*ass*_ − 1;

18:  **end while**

19:  Construct *CKC* ▹ Form *CKC* from all the *CKC*_*f*_ assigned and modified till now

20: **end for**

           ▹ **Local search phase**

21: Fix *L*      ▹ L is the maximum number of iterations

22: **for** (*l* = 0…*L*) **do**

23:  Select randomly a flight *f* such that |*CKC*_*f*_| ≥ 2;

24:  Remove a check-in counter from |*CKC*_*f*_|;

25:  Assign it to a flight in *X*_*f*_;

26:  Construct *CKC*;

27:  Measure *CKC*; ▹ Evaluate the solution associated to *CKC*

28: **end for**

29: Return the best *CKC*.

#### 3.1.3 Genetic heuristic

This heuristic is a standard genetic algorithm. Its main steps are reported in Algorithm 3. As already mentioned, the genetic procedure represents a reference case, since it is adopted by several simulators, such as arena [36] and Simul8 [37]. After generating the initial population of cardinality *S*, by randomly assigning the check-in counters to all flights, a cyclic subroutine selects two parents that will generate the new population, until a stop criterion is met. The probability of each individual to be selected for the reproduction is proportional to its fitness value. In such way the best individuals have a higher probability to transmit their genetic inheritance. The two operators that generate the new population from the parents are:

- The Crossover operator: it combines the genetic inheritance of the parents to generate new individuals. Each child is composed of two parts, each one belonging to one of the two parents. The *CR* is the rate of crossover. Each crossover operation generates two children.
- The Mutation operator: it randomly changes one or more components of an individual. The mutation operator is applied only with a certain probability, called mutation rate *MR*. The number of children that are generated by the mutation operator depends on the number of individuals generated by the crossover operator. The new population has the same cardinality of the initial one (*S*).

**Algorithm 3** Genetic heuristic

1: Given *I*    ▹ *I* is the maximum number of iteration of the algorithm

2: Given CR       ▹ CR is the rate of crossover

3: Generate *P* = {*p*_1_, *p*_2_, …*p*_*S*_}  ▹ The initial population of feasible solutions of cardinality *S*

4: **for** (*i* ≤ *I*) **do**

5:  **for** (*s* ≤ *S*) **do**

6:   Compute *Fitness*(*p*_*s*_)    ▹ The Fitness function corresponds the objective function of the problem

7:  **end for**

8:  Select the two parents *p*_*m*_ and *p*_*f*_ in *P* with the best Fitness function values

9:  Update p*p    ▹ * is the individual with the best Fitness function value

10:  *CC* = Crossover (*p*_*m*_, *p*_*f*_, *CR*);  ▹ *CC* is the set of individuals derived from the Crossover

11:  *M* = Mutation (*MR*) ▹ *M* set of individuals derived from the Mutation

12:  *P* = *CC* ∪ *M*

13:  *i* = *i* + 1

14: **end for**

15: Return p*

### 3.2 Simulation module

This section reports on the simulation parameters and the assumptions of the Simulation Module.

The model is a discrete-event simulation model: the state of the system changes when asynchronous events occur. Each event occurs at a particular time, thus the simulation can directly jump in time from one event to the next. The computation of the objective function value is possible thanks to the simulation of passenger flow, represented by the formation of lines at the check-in counters. The simulator also outputs the waiting time of the passengers in line.

The airport of Lisbon (LIS) is a valid case study to test the approach proposed in this paper. In fact, recent studies highlight that the access of passengers to the airport of Lisbon is strongly increasing year by year.

Moreover, regarding the check-in service, the 57% of the passengers use the counters to complete the check-in operations [38]. The airport of Lisbon is formed by two terminal areas. Terminal *T*1 is dedicated to the TAP Portugal international and charter flights. The departure floor of Terminal 1 hosts five check-in areas, in which 107 counters are located. This paper focuses on the optimization of Zone 3, hosting 69 check-in counters, but the same model and solution approach can be easily applied to the whole area or even to other terminal areas.

Given the airport layout, the passengers flow, the flights schedule and the time interval *T*, the simulation module emulates the passengers behavior and elaborates the value of the objective function, the passengers waiting times and lines length.

Considering the real passengers access demand to the airport of Lisbon during the year, it is possible to introduce the following representative assumptions:

- Discretization of the time: the time horizon *T* is divided into intervals (slots) of constant duration that is equal to 15 minutes. The system is represented by a discrete event model, and all the parameters and variables are referred to each slot *t* ∈ *T*.
- Flights distribution: the flights distribution in a 24h-day follows a normal distribution with parameters *μ* = 13 and *σ*^2^ = 8. In a day, different numbers of flights are considered for the tests, 75 to model low traffic, 105 for medium traffic and 130 for high traffic. The notation low, medium and high traffic refers to seasonal trend of the air traffic that is high during the summer, low during the winter and medium during the spring and the autumn.
- Arrivals distribution: check-in counter service demand can be expressed in terms of (a fraction of the) passengers arrival, represented by a stochastic variable. Two different cases have been tested. In the first case passengers arrive between 80 and 40 minutes before the scheduled departure time of the flight; in the second case passengers arrive between 100 and 20 minutes before the departure. In both cases, the expectation set to 60 minutes means that the most part of passengers arrive one hour before the departure. The first represents an homogeneous distribution of passengers per flight: the passengers arrival is characterized by a normal distribution with parameters *μ* = 60 and *σ*^2^ = 20; the second models a heterogeneous distribution: the passengers arrival is characterized by a normal distribution with parameters *μ* = 60; *σ*^2^ = 40.
- Passenger types: three types of passengers are here considered [39]: business (35%), travelling alone, with at most one baggage; tourist (40%), travelling alone or in groups up to 4 people carrying one (70%) or two (30%) luggage each and personal passengers (25%) with one bag.
- Service time: the service time represents the processing time of a passenger. The processing time at the check-in desk depends on the number of bags. It has a uniform distribution with variance of 20% and the following average processing times: no bags needs 1 minute, one bag needs 1.5 minute and 2 bags need 2.5 minutes.
- Number of check-in desks: the maximum number of check-in counters that can be opened is 69.

These assumptions are imposed to model a real case study able to test the solution approach, but it can be applied to any airport with any passenger flow distribution and type.

### 3.3 Cost function construction

The bi-criteria objective function considers two terms related to the operational costs and the passengers’ discomfort costs.

The latter has been constructed by implementing a pre-processing simulation phase that fits the cost coefficients associated to the waiting times to the standard Level of Service. It guarantees a balanced distribution of the cost in the two terms related to the opening of the check-in counters and to passengers’ discomfort. This issue is validated in the following analysis of the solution costs, that are similar in relation to the three heuristic procedures, and balanced in regard to the two cost items. The Eurocontrol defines the passenger value of time as an opportunity cost which corresponds to the monetary value associated with a passenger during a journey [40]. It describes, essentially, how much a passenger would be willing to pay in order to save time during a journey, or how much compensation he would accept, directly or indirectly, for the lost time. The passenger value of time varies according to the length, the purpose of the trip and the country, and the range goes from 13.6*e* to 77.8*e* per hour. The average opportunity cost, computed over all the categories of passengers, is around 50 euros/h, that is 0.8*e* per minute. Therefore, given the time intervals stated by the IATA, the step cost function derives from the average opportunity cost times the average value of each time interval. Moreover, the proposed cost function has been successfully compared to analogous cost functions reported in [40–42].

When considering the quality service standard defined by the [32], the unit costs to evaluate the objective function are fixed:

- the operational cost of a single check-in counter is equal to *C*_*o*_ = 20 euros/slot [39, 40, 43];
- the cost for the passengers’ waiting time *w*_*p*_ has been set to the following step function:
  $$\begin{array}{c} C_{p}\left(wt^{p}\right)=\{\begin{array}{cc} 0 & \text{if}\;w_{p}\leq 10\\ 15 & \text{if}\;11\leq w_{p}\leq 25\\ 25 & \text{if}\;26\leq w_{p}\leq 40\\ 40 & \text{if}\;41\leq w_{p}\leq 60\\ 80 & \text{if}\;w_{p}\geq 61 \end{array} \end{array}$$ (4)

## 4 Results and analysis

This section reports on the performance of the three algorithms.

As previously mentioned, usually check-in counters are divided into dedicated or common counters, depending on whether the desk is associated to a single flight or to a set of flights of a given company, respectively. A preliminary analysis has shown that the assumption of considering each check-in desk as common does not effect the qualitative analysis of the performance of the procedures [44]. When concerning the three different algorithms, the following notation has been used:

- *SM* stands for Surrogate Method Heuristic;
- *GRL* stands for Greedy heuristic plus local search;
- *GEN* stands for Genetic heuristic;

The results reported in the following are the average values calculated on 20 different runs with the same probability distribution of the simulation parameters.

### 4.1 Heuristics comparison

The comparison among the three heuristics concerns the computational times, the total cost function and its components, the average passengers’ waiting time and, finally, the average Level of Service guaranteed.

The mean computation times of the three procedures are comparable and amount to some seconds, with a peak of 12 seconds for the *GEN*.

**Fig 6:**
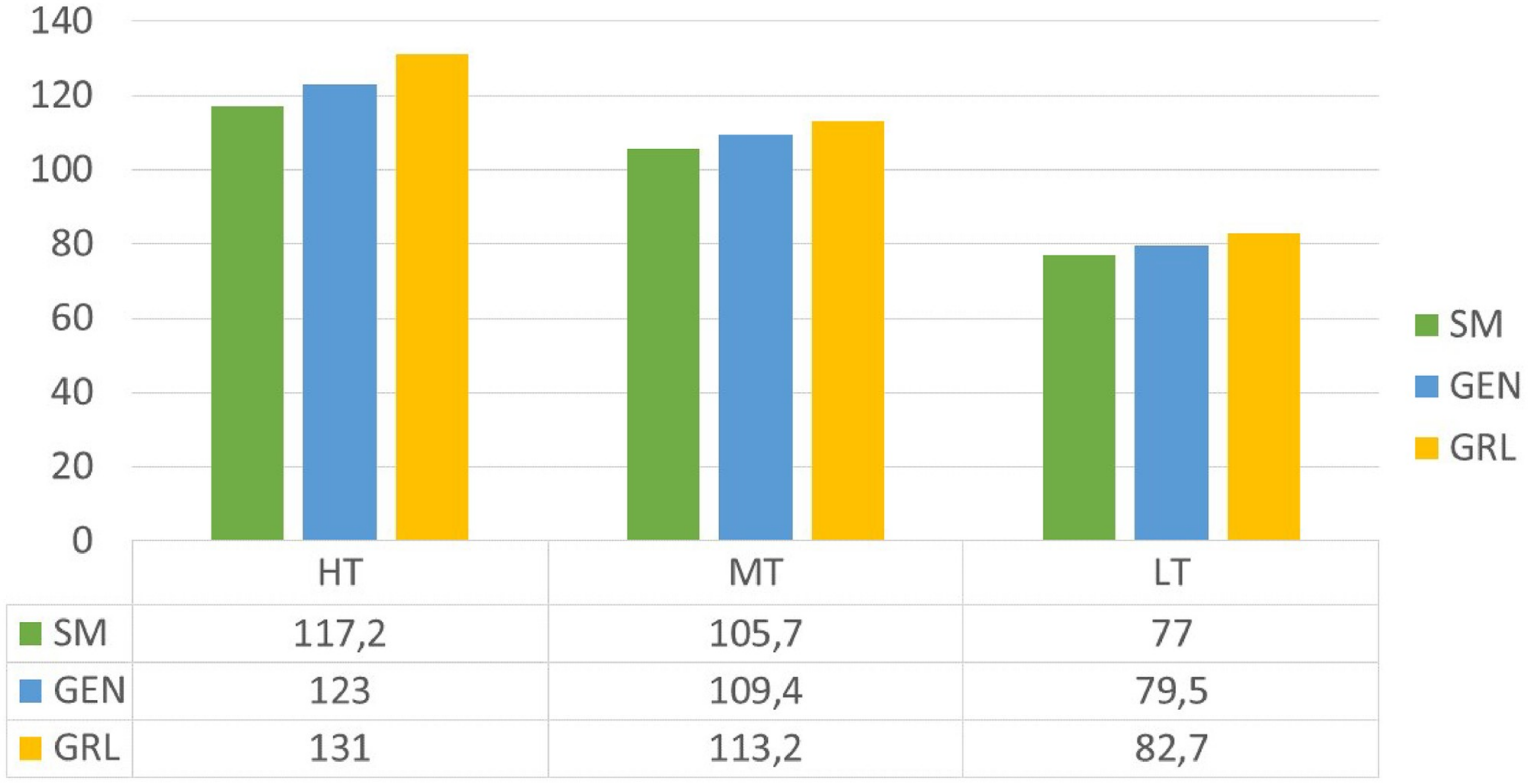
Total costs (K€): Operational costs + passengers’ waiting time costs.

In Fig 6 the total cost (K*e*) trend is shown. The *SM* outputs the best performances and these results confirm its ability to jump out of local minimum points of the objective function and to solve complex problems. When minimizing the total cost the improvement of the *SM* in relation to to the *GEN* is around 4.5% for all cases, considering all the flights in a day. Instead the improvement of the *SM* in relation to the *GRL* is around 8.5%.

In Table 2 the average passengers waiting time (in minutes) are reported for the different traffic scenarios. It is shown that the trends of *SM* and *GRL* are very similar. The *GRL* average improvement is around 2, 1% in relation to the *SM* and about 4, 3% in relation to the *GEN*.

**Table 2:** Average passengers waiting times (min).

Algorithm	*SM*	GEN	GRL
Low traffic	12.5	18	12.5
Medium traffic	12.83	20,75	12,5
High traffic	15.5	18.5	15

The service quality performed by the solutions is computed by considering the discomfort cost component of the objective function. Given the average waiting time for each passenger, the level of service of each solution is reported in relation to the International Air Transport Association standard.

**Fig 7:**
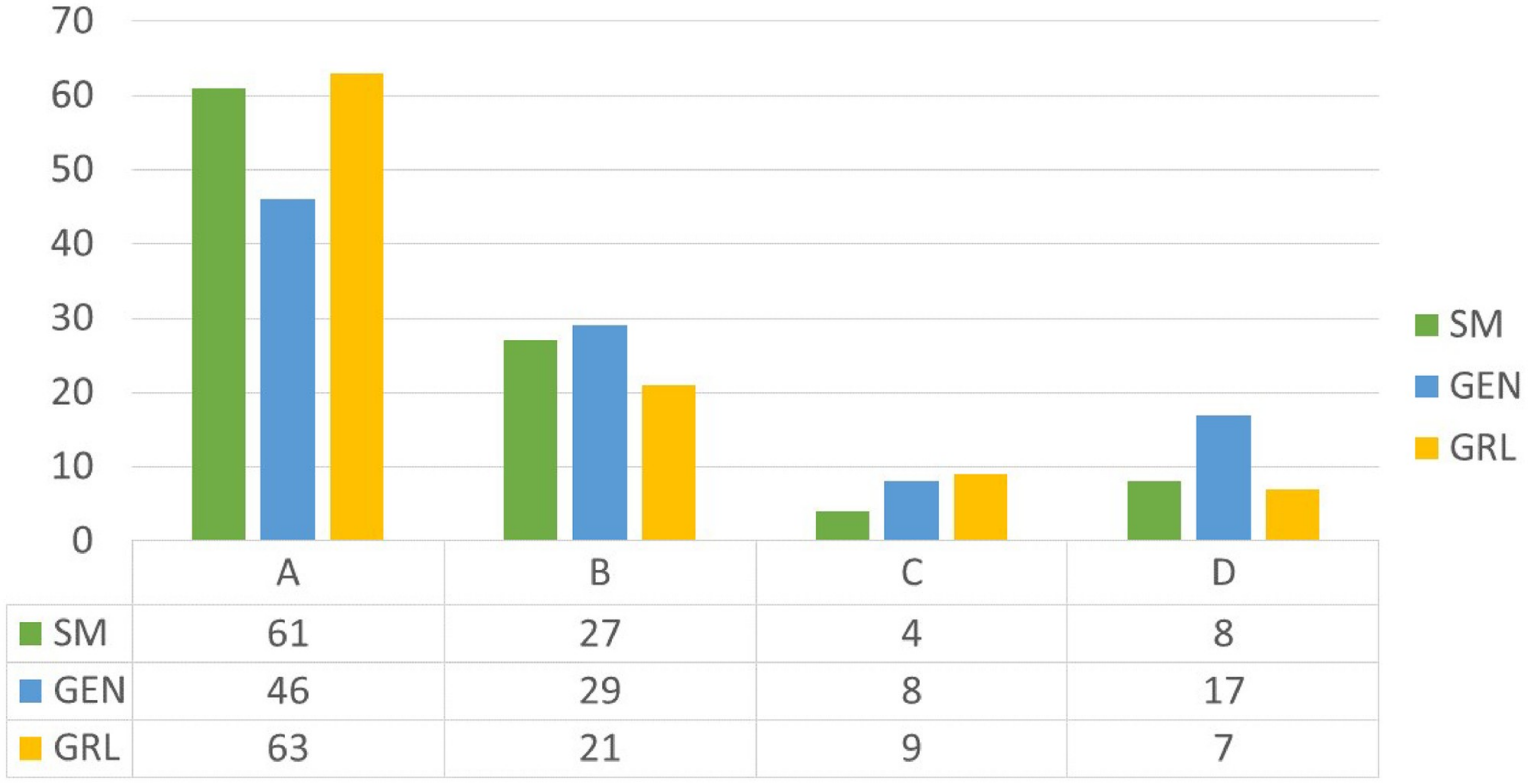
Service quality for low traffic and heterogeneous passenger distribution.

**Fig 8:**
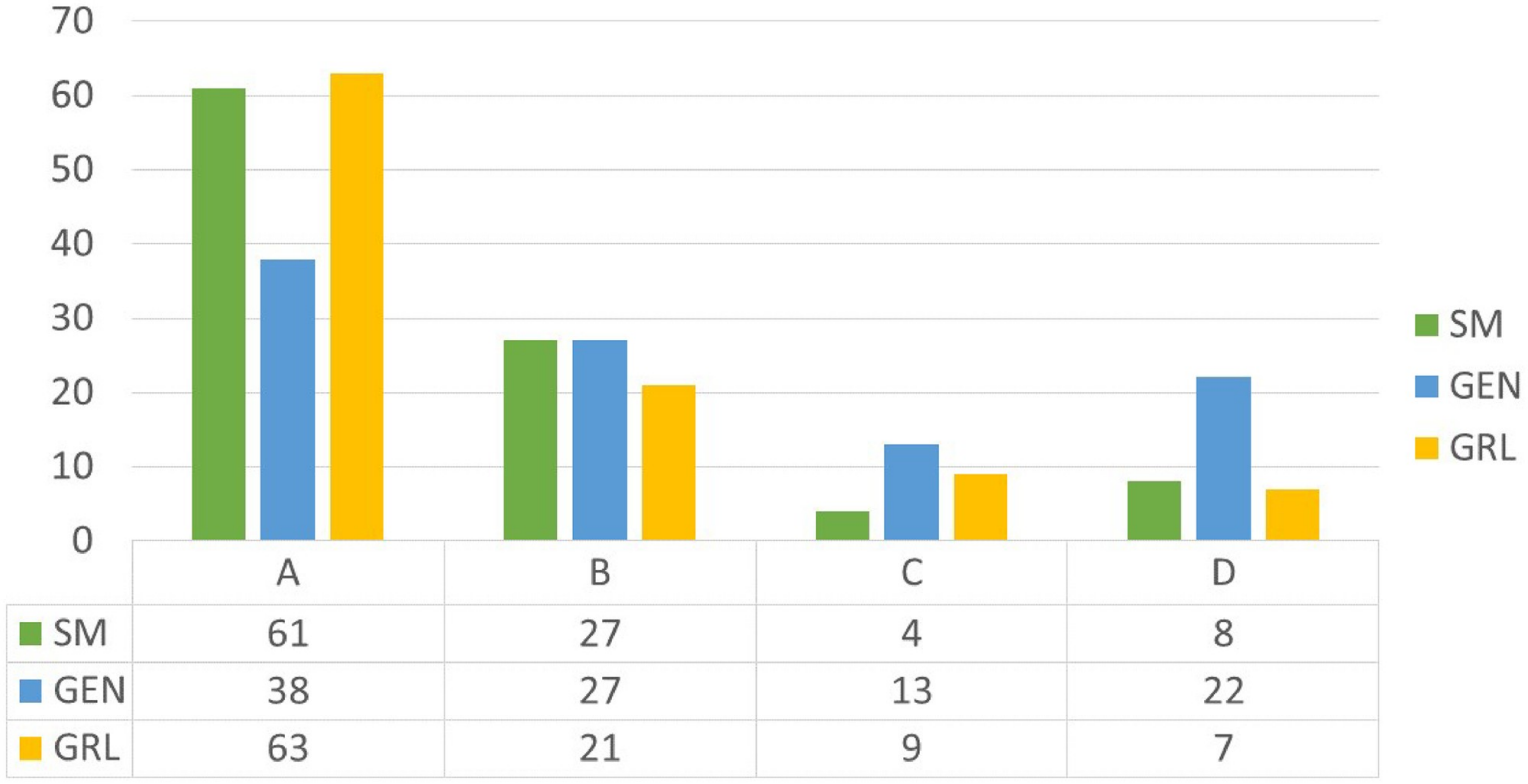
Service quality for medium traffic and heterogeneous passenger distribution (%).

**Fig 9:**
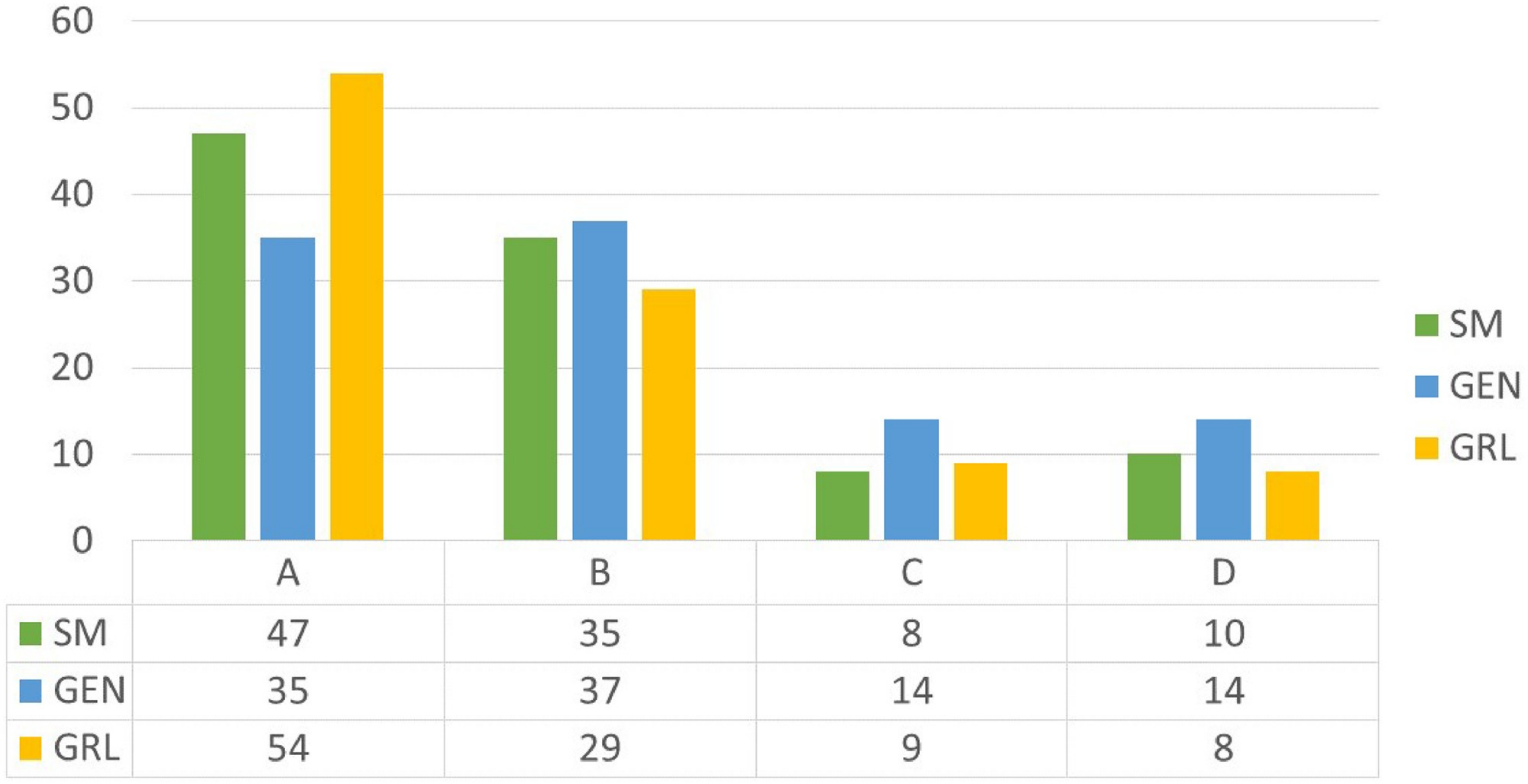
Service quality for high traffic and heterogeneous passenger distribution (%).

In Figs 7–9 the service quality of the solutions is reported with reference to the heterogeneous passengers’ distribution. The trend of the solutions is similar when considering the homogeneous case. As highlighted before, the *SM* gives the best performances in terms of total costs but when focusing on the service quality the *GRL* optimizes the passengers’ satisfaction inducing an even though negligible gap compared to the *SM*. When the traffic increases the *GEN* makes the service quality worse, in fact the majority of the solutions give a service quality equal to *B*. Instead for the *SM* and the *GRL* the highest percentage of the solutions persist in *A*.

In conclusion, the best results are given by the *SM* that properly combines the objective function value to the passenger satisfaction. On the contrary, the greedy heuristic focuses on the passenger satisfaction, neglecting the operational cost function. Concerning the *GEN*, it gives the worst performances both in relation to the total cost and to the service quality.

## 5 Conclusion

The increase of air transportation demand and the limited airports capacity call for the implementation of systems able to collect, analyse and process relevant data regarding passengers’ perception on ASQ.

In this paper authors address the problem of optimizing the check-in service problem for a set of flights in a given time interval. Besides the classical cost term related to the service operations, the objective function also models the passenger discomfort. Hence the Level of Service, based on the waiting time in line, is derived. The Surrogate Method has been tested in comparison with two other approaches: a Genetic Heuristic that is considered as benchmark (because of the most used procedure in literature to solve similar hard problems); and a Local Greedy Heuristic to favourite passengers satisfaction. The tests prove that *SM* gives the best performance in terms of total costs and of operational costs. Moreover it implements a Level of Service comparable to the best one given by the *GRL*. The real case of the Lisbon airport has been considered as the case study to highlight the performances of the *SM*, but the same approach can be applied to other terminal and passenger profiles.

The methodology presented in this paper could be integrated in a decision support system that can be shared both by the airline companies and by the airport quality management. In fact it is able to provide a check-in service schedule that perfectly balances the minimization of the companies operational costs and the maximization of the purpose of the airport management concerning the ASQ. In fact, this system provides recommendation about the subset of check-in desks to be opened during each time slot and which flights they have to process. In addition, the managers can use this system to focus their investigation on the perception of certain categories of passengers. The check-in service quality here derived can also be compared to the quality of other airport services in order to evaluate their ranking in the overall ASQ.

In future research the obtained results could be used to derive a *general* the Level of Service of the whole journey, from the entrance to the departure airport to the exit from the arrival airport, including all the involved process. Moreover, now that it has been proved the effectiveness of the implemented system in case of normal passengers flow and travelling policy, a straightforward evolution of this study could consist in adapting it to the new security restrictions that arise due to the Covid-19 pandemic. The authors retain that the system would be easily suitable to the new rules, since it can easily model in an appropriate way the capacity constraints, for example by decreasing the parameter related to maximum number of passengers in line to respect the spatial distances. The Level of Service of the solution can be easily deduced once new standard levels will be defined. In addition, it is well known that the pandemic has deeply affected the air transportation traffic demand causing a shocking decrease of it. Currently the new demand profile is unstable, hence an efficient, effective and robust decision support system that is able to react to the demand fluctuation, plays a critical role. Furthermore we can argue that when the Covid-19 emergency passes a transitory period will occur. In that case, a similar system can properly suit the demand variability and optimize the operational costs and the service quality.

## Supporting information

S1 File (ZIP)
